# Role of hypofractionated stereotactic radiotherapy for primary optic nerve sheath meningioma

**DOI:** 10.1093/nop/npad060

**Published:** 2023-10-03

**Authors:** İrem Koç, Sezin Yüce Sarı, Gözde Yazıcı, Yasemin Kapucu, Hayyam Kıratlı, Faruk Zorlu

**Affiliations:** Ocular Oncology Service, Department of Ophthalmology, Hacettepe University School of Medicine, Ankara, Turkey; Department of Radiation Oncology, Hacettepe University School of Medicine, Ankara, Turkey; Department of Radiation Oncology, Hacettepe University School of Medicine, Ankara, Turkey; Ocular Oncology Service, Department of Ophthalmology, Hacettepe University School of Medicine, Ankara, Turkey; Ocular Oncology Service, Department of Ophthalmology, Hacettepe University School of Medicine, Ankara, Turkey; Department of Radiation Oncology, Hacettepe University School of Medicine, Ankara, Turkey

**Keywords:** optic nerve sheath meningioma, radiosurgery, visual acuity, stereotactic radiotherapy

## Abstract

**Background:**

Optic nerve sheath meningiomas (ONSM) are rare tumors potentially causing visual deficits. This study aims to report the anatomic and visual outcomes of patients with primary ONSM treated with hypofractionated stereotactic radiotherapy (HF-SRT).

**Methods:**

Data of 36 patients treated with HF-SRT between 2008 and 2019 were retrospectively collected. The clinical target volume (CTV) was equal to the gross tumor volume and a 2 mm was added for the planning target volume. All responses other than progression were accepted as local control (LC). The VA grading was performed under 3 groups to provide an even distribution; 20/400 or worse, 20/40-20/400, and 20/40 or better.

**Results:**

Median HF-SRT dose was 25 Gy and the median CTV was 1.94 cc. After a median of 106 months of follow-up, the tumor regressed in 23 (64%), was stable in 9 (25%), and progressed in 4 (11%) eyes. The overall rate of LC was 89% with 2-, 5-, 10-, and 15-year rate of 100%, 94%, 84%, and 84%, respectively. Treatment-related late toxicity rate was 11%. The VA was stable in 27 (75%) eyes, improved in 5 (14%) eyes, and worsened in 4 (11%) eyes, respectively, after HF-SRT. Female gender was the only independent predictor of an improved VA.

**Conclusions:**

Hypofractionated stereotactic radiotherapy is a safe and satisfactory treatment option for primary ONSM without severe toxicity. It may be advisable to commence treatment before an established visual deficit of 20/400 or worse occurs, to make the most of the functional benefit.

Primary optic nerve sheath meningioma (ONSM) is a rare, benign tumor arising from the cap cells of the arachnoid villi surrounding the intraorbital portion of the optic nerve.^1^ It is the second most common intrinsic optic nerve tumor following optic glioma, and accounts for approximately 2% of all orbital tumors and 2% of all meningiomas.^2,3^ The treatment of ONSM is still controversial. The decision for treatment depends on several factors such as patient age, location of the lesion and visual acuity (VA). The primary aim of treatment is to preserve the VA, and treatment options include observation, surgery alone, surgery followed by adjuvant radiotherapy (RT), and RT alone.^4^ Active surveillance is an option for asymptomatic and nonprogressive lesions, whereas surgery is mainly of diagnostic value and has nearly been abandoned due to severe morbidity.^5^ Adjuvant RT following subtotal excision is an option but is not superior to both techniques per se.^6^ Therefore, RT is the treatment of choice for patients with symptomatic and/or progressive lesions.

Radiotherapy has particularly gained popularity in the last 2 decades owing to satisfactory results on long-term tumor control and also on immediate and long-term visual functions.^7^ However, the optimal RT technique is yet to be defined. Stereotactic radiosurgery (SRS) and fractionated stereotactic radiotherapy (FSRT) are novel treatment modalities, which treat the tumor in a spatial plane using precisely focused radiation beams in a single fraction and ≥2 fractions, respectively. Fractionated stereotactic radiotherapy has recently been used to refer to a hypofractionated (HF) RT scheme; however, it is mostly referred to as the stereotactic technique per se with conventional fractionation in the literature on ONSM and conventionally fractionated RT has been used in the majority of studies.

The literature on HF-SRT in the management of ONSM is limited. Herein, we aim to evaluate the clinical outcomes of HF-SRT in terms of tumor control with functional and visual results in patients with ONSM.

## Methods

### Patients

The records of patients with primary ONSM ≥16 years of age at diagnosis treated with HF-SRT between May 2008 and 2019 were retrospectively evaluated. All tumors were diagnosed via magnetic resonance imaging (MRI) findings. All patients were evaluated by a multidisciplinary tumor board prior to treatment. As a general approach at our facility, the indication for treatment is radiological or functional deterioration, and only severely debilitated cases with a VA of 20/400 or worse were offered HF-SRT upon first admission. Patients with a secondary OSNM and patients with a history of a prior treatment were excluded from the study. The study was approved by the Hacettepe University Non-Interventional Clinical Research Ethics Committee (Protocol no. 19/832) and carried out in accordance with Declaration of Helsinki.

### Hypofractionated Stereotactic Radiotherapy Technique

**Figure 1.**
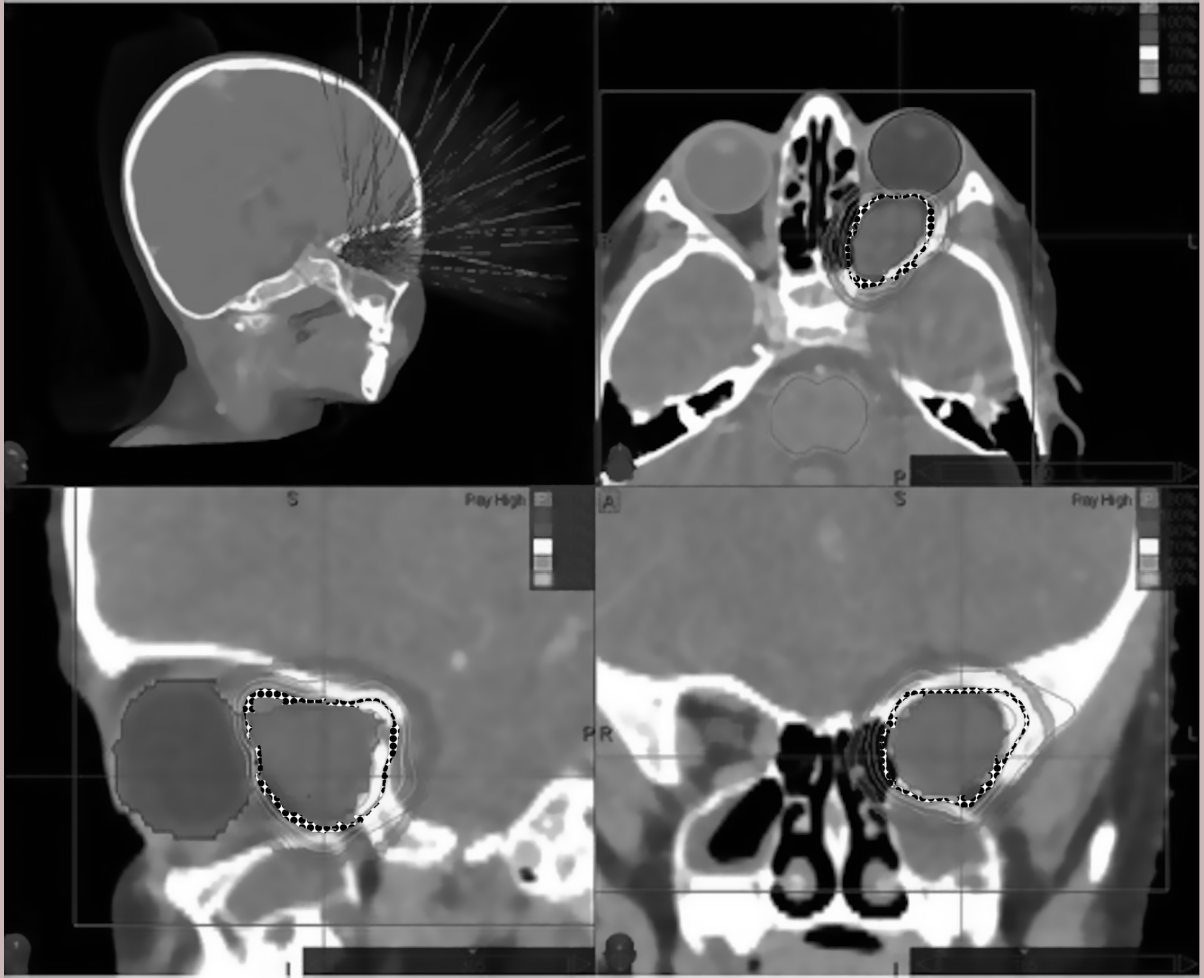
The CK plan of a patient with primary ONSM surrounding the left optic nerve. The left-upper image shows the active and passive beams directed to the PTV. The right upper, left lower, and right lower images shows the axial, sagittal, and coronal sections of the planning CT, respectively. The dose is prescribed to the 80% isodose shown in an dotted interrupted line around the PTV.

Patients underwent HF-SRT via CyberKnife (Accuray) or Novalis (BrainLab). Patients were treated in supine position and a thermoplastic mask was used for immobilization. A slice thickness of 1 mm was obtained for both planning computed tomography (CT) and MRI. For each patient, the target volume and organs at risk (OARs; ie, bilateral eyes, lenses, optic nerves, and the optic chiasm) were delineated using the fused CT/MRI data set. The gross tumor volume (GTV) was defined as the contrast-enhanced lesion visible in the fused CT/MRI image. The clinical target volume (CTV) was equal to the GTV, and the planning target volume (PTV) was defined as the CTV plus a safety margin of 2 mm in all directions. An individualized treatment plan was developed for each patient. Treatment plans were optimized according to the requirement that ≥99% of the PTV received 95% of the prescribed dose, taking into account the dose constraints of OARs. The target volume delineation and CyberKnife plan of a patient are shown in Figure 1. All patients were treated on consecutive weekdays and completed their planned treatment schedule without any interruption.

### Follow-Up and Toxicity

Tumor response was assessed via ophtalmologic examination, MRI, and endocrine tests 12 weeks after HF-SRT was completed. All patients were followed up and examined every 3 months during the first 2 years, and annually thereafter. Follow-up information was obtained from the department charts, hospital notes, referring doctors, and from patients and/or next of kin. The incidence of acute and late toxicity was also noted.

Notable systemic conditions, initial complaint at admission, initial and final Snellen VA with initial and final tumor dimensions on the MRI were collected where available. Tumor dimensions were expressed as D1 (axial) and D2 (sagittal), and were measured on the largest measuring cross-section on the MRI. As an outcome measure, radiological progression was defined as any increase in D1 or D2 in at least 2 consecutive MRI scans, stable disease was considered if both remained stable, and regression was considered in case of any decrease in either parameter. All responses other than progression were accepted as local control (LC). The variations in D1 and D2 were also separately recorded; a positive value at the last control was accepted as an increase and a negative value as a decrease in the dimension size, respectively. Because meticulous best-corrected VA testing was not repeated at every visit but the refractive error was corrected according to the previous prescription, the VA grading was performed under 3 groups to provide an even distribution. These groups were: (1) 20/400 or worse, (2) 20/40-20/400, and (3) 20/40 or better.

### Statistical Analysis

All statistical analyses were performed using the Statistical Package for the Social Sciences (SPSS) version 23.0 (SPSS Inc.). Statistical analyses included descriptive statistics indicating median, minimum, and maximum values for data not showing normal distribution. Kruskal–Wallis test was performed to test differences in continuous variables among groups of different morphology. McNemar-Bowker test was employed to see if frequency distribution of the VA was different before and after treatment. A multiple linear regression model was used to identify independent predictors of the VA. The model fit was assessed using appropriate residual and goodness-of-fit statistics.

The primary end point was LC and secondary end points were treatment-related toxicity and variation in VA. The survival analysis was carried out using the Kaplan–Meier method and was compared using the log-rank test. Age (≤45 vs >45 years), gender, D1 at diagnosis (≤20 vs >20 mm), D2 at diagnosis (≤15 vs >15 mm), radiological morphology (tubular vs fusiform vs globular), intraorbital extension, intracanalicular extension, intracranial extension, VA at diagnosis (worse than 20/400 vs 20/40-20/400 vs better than 20/400), total HF-SRT dose (≤22.5 vs <22.5 Gy), CTV size (≤3 vs >3 cc), and HF-SRT device (CyberKnife vs Novalis) were included in the univariate analysis. Multivariate analysis was planned to be performed using the Cox proportional hazards model, using all factors with a *P* ≤ .1 by univariate analysis. For all other measures, *P* < .05 was considered statistically significant.

## Results

This retrospective analysis included 36 patients who underwent HF-SRT for primary ONSM in one eye only. Thirty-two (89%) patients were female and 4 (11%) were male. The median age at diagnosis was 45 years (range: 17, 77 years). The most common complaint in the affected eye was progressive loss of vision and proptosis, whereas 4 eyes (11%) were incidentally diagnosed. The most common comorbidities in patients were hypertension and coronary artery disease. Morphologically, 16 (44%) tumors were tubular, 11 (31%) were fusiform, and 9 (25%) were globular. The most common optic disc findings at the first examination were pallor (*n* = 16, 44%) and edema (*n* = 6, 17%) whereas 3 (8%) eyes had normal optic disc findings. The median D1 and D2 was 20 mm (range: 6, 32 mm) and 13 mm (range: 6, 27 mm) at diagnosis, respectively. The VA at diagnosis was worse than 20/400 in 18 (50%), 20/40-20/400 in 12 (33%), and 20/40 or better in 6 (17%) eyes, respectively. Intraorbital, intracanalicular, and intracranial extension was present in 19 (53%), 13 (36%), and 11 (31%) eyes, respectively.

Twenty-nine (81%) patients were treated via CyberKnife and 7 (19%) were treated via Novalis. Median total dose of HF-SRT was 25 Gy (range: 20, 25 Gy) and median dose per fraction was 5 Gy (range: 4, 7.5 Gy) in median 5 (range: 3–5) fractions. Median CTV was 1.94 cc (range: 0.38, 22.68 cc). The detailed characteristics of the HF-SRT plans are shown in Table 1.

**Table 1.** Characteristics of HF-SRT Plans for ONMS

Characteristic	Median value (range)
HI	1.16 (1.02–1.30)
CI^*^	1.54 (1.11–3.91)
Collimator size^*^	10 mm (5–20 mm)
Normalization isodose^*^	85% (77%–90%)
*N* of beams^*^	184 (106–330)
Maximum dose to the CTV	27.78 Gy (25–32 Gy)
Maximum dose to the ipsilateral eye	24.6 Gy (4.6–28.5 Gy)
Maximum dose to the contralateral eye	5.1 Gy (0.3–14.1 Gy)
Mean dose to the ipsilateral lens	6.9 Gy (1.6–13.3 Gy)
Mean dose to the contralateral lens	3.6 Gy (0.1–10.4 Gy)
Maximum dose to the ipsilateral ON	25.6 Gy (5.8–30.2 Gy)
Maximum dose to the contralateral ON	5.9 Gy (0.4–25.8 Gy)
Maximum dose to the optic chiasm	10 Gy (3.2–25.8 Gy)

CI = conformity index; CTV = clinical target volume; HF-SRT = hypofractionated stereotactic radiotherapy; HI = homogeneity index; *N* = number; ON = optic nerve; ONMS = optic nerve sheath meningioma.

^*^For patients treated with CyberKnife only.

**Figure 2.**
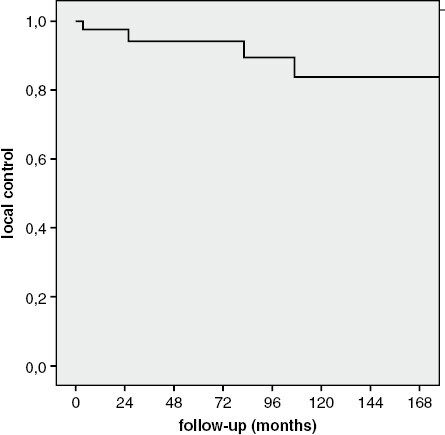
Local control rate during follow-up.

Median follow-up was 106 months (range: 23, 185 months), and all patients were alive at the time of analysis. The median D1 and D2 at the last control was 16 mm (range: 3, 35 mm) and 12 mm (range: 4, 26 mm), respectively. The median variation in D1 was 4 mm (range: −3, 12 mm) and in D2, it was 0 mm (range: −3, 16 mm). Following HF-SRT, the tumor regressed in 23 (64%), was stable in 9 (25%), and progressed in 4 (11%) eyes, respectively. The overall rate of LC was 89%. The 2-, 5-, 10-, and 15-year LC rate was 100%, 94%, 84%, and 84%, respectively (Figure 2). In univariate analysis, no prognostic factors were found for LC. Therefore, multivariate analysis could not be performed.

No treatment-related acute toxicity was detected. Overall distribution of the VA was similar in pre- and posttreatment comparisons (*P* = .083). The VA classification was stable in 27 (75%) eyes, improved in 5 (14%) eyes, but worsened in 4 (11%) eyes, respectively, at the last control compared to the first visit. In 4 patients whose tumors radiologically progressed, the VA was stable at the last control. In 5 eyes with an improved VA, the tumor regressed in 3 and was stable in the other 2. In 4 eyes with a worsened VA, the tumor regressed in 2 and was stable in the other 2. Therefore, treatment-related VA worsening was seen in 4 patients, and the rate of late toxicity was 11%. No significant relation was found between the tumor response to HF-SRT and the VA (*P* = .49). In univariate analysis, male gender and lower total HF-SRT dose were found as negative significant prognostic factors for the VA. Besides, D2 at diagnosis, intracanalicular extension and lower maximum dose to the ipsilateral eye also deteriorated the VA outcomes (Table 2). In multivariate analysis, the only independent predictor of a better VA following HF-SRT was female gender (*P* = .02).

**Table 2.** Prognostic Factors for the Final VA

Factor	Final VA *N* of patients (%)			*P* value
	Improved	Stable	Worsened	
Gender				
Female	5 (16)	26 (81)	1 (3)	<.001
Male	0 (0)	1 (25)	3 (75)	
D2 at diagnosis				
≤1.5 cm	2 (9)	20 (91)	0 (0)	.085
>1.5 cm	3 (30)	6 (60)	1 (10)	
Intracanalicular extension				
Absent	5 (20)	19 (76)	1 (4)	.051
Present	0 (0)	8 (73)	3 (27)	
Total HF-SRT dose				
≤22.5 Gy	2 (13)	9 (60)	4 (27)	.041
>22.5 Gy	3 (14)	18 (86)	0 (0)	
Maximum dose to ipsilateral eye				
≤25 Gy	4 (21)	11 (58)	4 (21)	.07
>25 Gy	1 (7)	13 (93)	0 (0)	

HF-SRT = hypofractionated stereotactic radiotherapy; *N* = number; VA = visual acuity.

## Discussion

In the current study, we achieved an overall LC rate of 89% following HF-SRT to patients with primary ONMS after a median follow-up of more than 8 years. The rate of HF-SRT-related late toxicity was 11%, but the VA was stable or improved in 89% of patients. Female gender was associated with a significantly worse VA.

The management of primary ONSM was mainly restricted to observation until nearly a decade ago because of concerns from severe complications due to the close proximity of the tumor to the optic nerve, and high recurrence rates with surgery.^3,8^ Even though life expectancy is excellent, observation without treatment eventually almost always leads to visual deterioration in the long run and there is an up to 38% risk for the contralateral eye to be affected.^3,4,9^ Kennerdell et al.^8^ recommended to initiate treatment when the VA progressively worsens to the level of below 20/40 or the visual field is constricting. Since the early 2000s, RT for ONSM has emerged as a treatment option and gained popularity with satisfying radiological and visual outcomes compared to surgery.^10^ The literature on RT for ONSM presents different views on this issue. Adjuvant RT after debulking surgery is an option with up to 67% stable and 44% improved vision rates.^6,8^ However, this combination does not decrease postoperative complications compared to total excision alone. Selected patients with large tumors and useful vision may benefit from this combination.

Smith et al.^11^ were the first to publish the results of RT alone in primary ONSM. The effective conventional RT dose for tumor control was around 50–60 Gy in the first studies.^8,12,13^ Dutton^3^ reported the VA was improved in 75% and stable in 8% in a review of 12 patients from the literature. When observation, surgery, RT, and surgery with adjuvant RT were compared, the best long-term visual outcomes were observed with RT alone at the expense of treatment toxicity in one-third of the patients.^6^ In addition to this concern of severe RT-related toxicity, the belief that meningiomas are radioresistant prevented RT to be the treatment of choice for ONSM for years.

Later, with the increasing use of SRT techniques, RT has become popular owing to a more homogeneous dose distribution and better sparing the OARs. A most recent review compared the oncologic and functional outcomes of different RT techniques and found no significant difference among conventional RT, 3-dimensional conformal RT (3DCRT), intensity-modulated RT, SRS, FSRT, and proton beam therapy.^14^ However, the definitions of SRS and FSRT in this review were different from the traditional definitions. The number of fractions in the 19 studies of FSRT was between 26 and 30, and in the 4 studies of SRS, it was 2, 3, 4, and 5, respectively. Therefore, when the outcomes of SRS studies, which actually performed FSRT were assessed, median follow-up time was 32 months (20–56 months), the overall LC rate was 90.6% (80%–100%) in a total of 59 eyes, and the VA improved in a mean 52% (20%–100%) of them. RT-related toxicity was observed in a mean 4.5% (0–4.5%) of these patients which in all was optic neuritis solely. The toxicity rate with SRS was significantly lower compared to 3DCRT but similar to other techniques.

In 2021, Senger et al.^15^ reviewed the results of 5 studies on 87 patients with ONSM treated with FSRT or SRS via CK. The total treatment dose ranged between 14 and 25 Gy in 1–5 fractions, and the overall LC rate between 93% and 100%. In the 27 eyes of their own 25 patients, the authors reported the LC rate 96% with 20–25 Gy in 4–5 fractions. The VA was stable in 90% and improved in 10% of the eyes after treatment without a significant difference compared to the VA prior to treatment. Furthermore, the treated volume and the dose to the ipsilateral optic nerve did not have an impact on the variation in the VA. It is important to note that one-third of the eyes in this study had undergone surgery prior to RT. An Israeli study compared the outcomes of conventionally fractionated RT of 50.4 Gy in 28 fractions (*n* = 35) to HF-SRT of 22.5–30 Gy in 5 fractions (*n* = 13) for anterior visual pathway meningiomas.^16^ Although the visual functions were better after conventional RT, the authors reported no statistically significant difference between the 2 RT techniques with regard to the VA and radiologic response.

The close vicinity of the tumor to the optic pathway was the main concern for avoiding SRS for a long time, and it was even accepted contraindicated when the distance between the tumor and the anterior visual pathway is less than 3 mm.^17–19^ However, SRS can safely be performed on these tumors as well. Conti et al.^20^ reported the outcomes of 64 patients with perioptic meningioma located within 2 mm of the optic apparatus. All patients were treated with CK. The data of the first 25 patients were given retrospectively, who were treated in 2–5 fractions to a total 18–25 Gy. After a median follow-up of 57.5 months, the LC rate was 100% without any deterioration in the VAs. The authors then treated 39 patients and gave the prospective outcomes of 18–40 Gy in 2–15 fractions. After a median of 15 months, the LC rate was again 100% and no VA deterioration was observed. The authors concluded that a biologically effective dose in 2-Gy equivalents (BED_2Gy_) > 100 Gy results in a better tumor control, and the risk of optic neuropathy can be minimized by keeping the dose to the optic pathway <5 Gy per fraction.

A Taiwanese study also reported the retrospective results of FSRT in 60 patients with perioptic meningioma or schwannoma with lesions of ≤3 mm close to the optic apparatus.^21^ In the 57 patients with meningioma, only 14 were de novo and underwent definitive FSRT. The median tumor volume was 7 cc, and the authors did not give any safety margin to the GTV for the PTV. All patients were treated with 3 fractions of 6–7 Gy, and the mean dose to the optic pathway was 6 Gy. After a median follow-up of 52 months, the tumor volume decreased in 8 (15%) patients and the overall LC rate was 87%. The authors reported that the volume increased in 7 (12%) patients; however, they defined “increase” when there was at least a 20% increase in the tumor volume. Hence, the real LR rate would be higher according to our definition of progression. While the VA improved in 5 (8%) patients, it worsened in 6 (10%) patients with meningioma in that study, but no optic neuropathy was observed. Although the median GTV in this study seems larger than ours, the PTV-margin of 2 mm in our study makes our PTV volumes much larger. Therefore, 18–21 Gy in 3 fractions for small ONSM does not seem adequate.

The recommended dose for SRS in meningiomas is 14 Gy which has a BED_2Gy_ of 79.33 Gy when α/β is 3.^22^ However, this cannot be accomplished for ONSM. The maximum dose to the optic pathway is closely related to the risk of optic neuropathy and volumetric constraints are less important.^23^ Tishler et al.^19^ reported a risk of 24% with >8 Gy in a single dose to any portion of the optic pathway. On the other hand, Leber et al.^17^ did not observe optic neuropathy with <10 Gy, but the rate was 27% for 10–15 Gy and 78% for >15 Gy, respectively. Moreover, Mayo et al.^24^ reported that the risk was significantly increased with >12 Gy in a single fraction. Therefore, with a higher recommended dose for meningiomas, SRS does not seem to be the optimal RT technique.

Fractionated schemes can help increase the dose to the tumor and the optic pathway can also receive a higher total dose without an adverse event.^20,25,26^ While the QUANTEC recommends the maximum dose to the optic pathway 12 Gy for a single fraction,^24^ Timmerman^27^ reported 19.5 Gy in 3 fractions safe and the AAPM Task Group 101 reported 25 Gy in 5 fractions resulted in <1% risk for optic neuropathy.^28^ These recommendations were also justified in Hiniker et al.’s study.^23^ Besides, the estimated risk was 1.3% for 22 Gy and 1.9% for 24 Gy, respectively, in 3 fractions, whereas the respective doses were 27.5 Gy and 30 Gy in 5 fractions. Likewise, the accepted doses to the optic pathway were 10 Gy in 2 fractions, 15 Gy in 3 fractions, 20 Gy in 4 fractions, and 25 Gy in 5 fractions in the study of Conti et al.,^20^ which are all in the range of our limits. The maximum dose to the optic pathway was a median of 25 Gy in our study, and we did not observe any acute toxicity related to HF-SRT, most likely related to the much smaller fields and a safer scheme in 3–5 fractions. Knowing the fact that the onset of optic neuropathy may prolong to 9 years, 11% rate of late optic neuropathy may increase in our patients in the following years.^29^

There are certain limitations to our study, mainly due to its retrospective nature and relative rarity of the disease. First, the patients did not have a pathological confirmation, and the diagnosis of ONMS was based on imaging findings. However, this is also true for most studies in the literature on definitive RT. Secondly, the VA is only a compound of visual function and the effects of HF-SRT on visual functions should be assessed in a broader sense. For this purpose, newly acquired imaging modalities such as optical coherence tomography angiography could also be incorporated to assess the optic nerve head and macular blood flow, which are crucial anatomical landmarks for VA. However, our study showed that HF-SRT for primary ONSM is functionally and to a greater extent anatomically safe and effective method of treatment, irrespective of tumor morphology. With the overall LC rate of 89% and a late complication rate of 11%, 20–25 Gy in 3–5 fractions can be the treatment of choice, even for tumors very close to the optic pathway. We hope the ongoing single-arm prospective trial would give us better data on the treatment of primary ONSM which are in close proximity to the optic pathway.^30^ Commencing treatment before a visual deficit of 20/400 or worse occurs would be preferred as HF-SRT improved the VA in only 14% of the eyes.
